# Corn Crisps Enriched in Omega-3 Fatty Acids Sensory Characteristic and its Changes During Storage

**DOI:** 10.1007/s11746-016-2873-y

**Published:** 2016-07-23

**Authors:** Mateusz Rogalski, Karolina Nowak, Piotr Fiedor, Arkadiusz Szterk

**Affiliations:** 1 Faculty of Human Nutrition and Consumer Sciences, Warsaw University of Life Sciences, 159 c Nowoursynowska, 02-776 Warsaw, Poland; 2 National Medicines Institute, 30/40 Chełmska, 00-725 Warsaw, Poland; 3 Department of Spectrometric Methods, National Medicines Institute, 30/40 Chełmska, 00-725 Warsaw, Poland

**Keywords:** n-3 Fatty acids, Functional food, Pro-health food, Argon, Modified atmosphere packing

## Abstract

Extruded cereal snacks are usually deficient in protein, mineral ingredients, valuable fatty acids. With the rise of health awareness among consumers, food manufacturers and scientists are pressed to take measures in order to develop new functional/health-beneficial foods. The aim of this work was to manufacture extruded crisps enriched with α-linolenic acid (obtained from linseed oil) and to observe whether storage of the product for the period of 6 months would cause its disqualification, primarily due to its sensory properties and secondarily due to its chemical properties. The research demonstrated that the addition of linseed oil to corn crisps at the amount of 5 % enables to obtain functional corn crisps containing over 2 g of ALA in a portion of 100 g even after 6 months of storage at room temperature. ALA-enriched crisps maintain the original sensory profile after 6 months of storage and their sensory profile is similar to the profile of crisps without the addition of linseed oil if they are packed in barrier packaging filled 100 % with argon. Therefore, they may be a healthier alternative to typical corn crisps.

**Electronic supplementary material:**

The online version of this article (doi:10.1007/s11746-016-2873-y) contains supplementary material, which is available to authorized users.

## Introduction

Extruded products are usually characterized by high energy content accompanied by low nutritional value: small amounts of vitamins, mineral ingredients, fiber, polyunsaturated fatty acids [1, 2]. Extrusion is widely used to produce salty and sweet snacks as well as breakfast products which are commonly consumed by people around the world. Owing to the relatively widespread production of extruded foods, scientists undertake technological endeavors in order to increase their nutritional value [3, 4]. One of the methods of increasing the nutritional value of extruded products is their enrichment with polyunsaturated omega-3 fatty acids (n-3), e.g. α-linolenic acid (ALA). The process of enrichment of food with n-3 fatty acids (including ALA) is a very important issue which is a subject of interest among many scientists all over the world [5–7]. It is associated with numerous beneficial health effects of n-3 acids on the functioning of a human body. ALA is a very important precursor to the synthesis of other n-3 fatty acids such as eicosapentaenoic acid (C20:5n3: EPA), docosapentaenoic acid (C22:5n3: DPA) as well as docosahexaenoic acid (C22:6n-3: DHA). A human body can produce up to 5 % of EPA and DHA due to enzymatic conversion of ALA into these fatty acids [8, 9]. ALA and its metabolites play a very important role in the appropriate growth and development of humans and animals. ALA, EPA and DHA are all very significant fatty acids which are necessary for the proper growth of children and have to be supplied to the human body through diet [10, 11]. ALA plays a very important preventive role and helps to fight cardiovascular diseases [12], cholesterolemia [13], it decreases the risk of myocardial infarction [14], as well as stroke [15, 16]. The best source of ALA is linseed oil which on average contains 60 % of ALA in the fatty acids profile, but is also commonly observed in other food products, although in considerably lower concentration, for instance edible plants seeds such as colza oil (up to 10 %) or in perilla seeds, walnuts, chia and chloroplasts in green parts of plants [17–19]. According to the European Union Law, namely the regulation of European Commission No. 432/2012 of 16 May 2012 (with later amendments) [20], functional/health-beneficial ALA-rich corn crisps may help in maintaining an appropriate level of cholesterol in blood and may also come as a valuable source of omega-3 fatty acids if the human body receives 2 g of ALA through their consumption within a day. Our prior studies have demonstrated that a 5 % addition of refined linseed oil to corn grit allows the production of functional corn crisps containing 2.6 g of ALA per 100 g^−1^ in a complete product [19, 21]. However the addition of various lipids such as triacylglycerols, ethyl esters or free fatty acids plays a crucial role in extrusion cooking for instance significantly affects the expansion during the extrusion process as well as the other physical properties of corn crisps like porosity, hardness, shape etc. [19]. Our previous research proved that the addition of triacylglycerols (TAG) obtained from linseed oil as a source of n-3 fatty acids is the best recipe-technological approach from textural properties point of view [19, 21]. In that case the structure and expansion are similar to those of typical corn crisps without any lipid enrichment [19]. The addition of polyunsaturated fatty acids to extruded products will contribute to the intensification of unfavorable oxidation processes [21]. This is caused by low water activity and highly developed interphase surface resulting in a heavily porous material [19, 21, 22]. The amount of fat exceeding 2.5 % significantly impacts the oxidation stability of extruded products, especially those enriched with oxidation unstable polyunsaturated fatty acids [1, 23, 24]. Oxidation changes of the lipid fraction in extruded products result in a change of flavor. Products rich in polyunsaturated fatty acids have to be protected against such changes. Various studies around the world contain a multitude of data concerning food enrichment with n-3 fatty acids as well as the chemical changes of these acids. However, very few scientists have conducted an in-depth research concerning the sensory changes, which naturally determine the success of a product on the market. In the case of functional/health-beneficial foods this aspect is as important as the chemical structure of these products. In most cases, manufacturing a food product rich in active biological ingredients does not constitute a technological problem. However, manufacturing a functional product which would comply with, e.g., the regulation of European Commission No. 432/2012 [20] and at the same time would taste good is a challenge for scientists and food technologists. It is known that sensory properties of food change during its storage because of various chemical, microbiological and/or biochemical reactions that occur over that time. A functional product has to meet two basic criteria. It has to possess an appropriate chemical composition (in this case at least 2 g of ALA per portion on the expiry date) and maintain the same flavor at the end of the best-before period. There have been no studies concerning the sensory properties of corn crisps enriched with n-3 fatty acids of plant origin. There is a need for such research in order to obtain a complete profile of functional corn crisps.

The aim of this work was to study quantitative and qualitative sensory profiles as well as the general quality of various functional corn crisps enriched with α-linolenic acid which were stored for 6 months in barrier packages filled with different gases. The research goal was to determine whether it is possible to manufacture functional corn crisps rich in ALA while maintaining high sensory quality, namely the same or higher quality in comparison to typical corn crisps without the addition of linseed oil. A comprehensive sensory analysis and its association with the chemical composition allows for an adequate assessment of a functional product which has to comply with chemical, technological, and sensory requirements. Otherwise, we cannot treat it as functional food since nobody would consume it because of its bad taste/flavor.

## Materials and Methods

### Chemicals

Heptane CHROMASOLV^®^ Plus, for HPLC, assay 99 % (product number: 650536), chloroform anhydrous, assay ≥99 %, contains 0.5–1.0 % ethanol as stabilizer (product number: 288306), methanol CHROMASOLV^®^, for HPLC, assay ≥99.9 % (product number: 34860), sodium hydroxide reagent grade, assay ≥98 %, pellets (anhydrous) (product number: S5881), boron trifluoride-methanol solution (BF_3_-methanol solution) 14 % in methanol (product number: B1252), methyl undecanoate certified reference material, TraceCERT^®^ (product number: 47147). All chemicals were bought from Sigma Aldrich, Poznan, Poland.

### Research Material

The research material extruded corn crisps obtained from corn grit with or without the addition of preparation from refined linseed oil (RLO), RLO and natural antioxidants such as δ-tocopherol. The corn crisps were obtained exactly as described by Szterk *et al*. [19]. The extrusion was carried out under industrial conditions in a factory belonging to Zakład Produkcyjny Stodzew, Stodzew 54, 08-441 Parysów, Poland (Grupa BGK Sp. z o.o. Poland).

The following types of corn crisps were produced (40 kg of each type):

- Control: corn crisps produced only from corn grits.
- TAG: corn grits with the addition of 5 % RLO.
- EE: corn grits with the addition of 5 % fatty acid ethyl esters. Fatty acid ethyl esters were produced according to Szterk *et al*. (2015) [19].
- FFA: corn grits with the addition of 5 % free fatty acids. Fatty acids were produced according to Szterk *et al*. [19].
- TAG 200: corn grits with the addition of 5 % RLO which contained the addition of 200 mg 100 g^−1^ of δ-tocopherol.
- TAG 500: corn grits with the addition of 5 % RLO which contained the addition of 500 mg 100 g^−1^ of δ-tocopherol.
- TAG 800: corn grits with the addition of 5 % RLO which contained the addition of 800 mg 100 g^−1^ of δ-tocopherol.

Each type of the crisps produced was divided into two parts. One part was packed in atmospheric air, while the other was packed in 100 % argon gas. The packing process was performed using an EDESA VAC-10S packing machine, and applying the following packing program:

The crisps were packed in PA/PE barrier film protective packaging: the thickness of layers was 30/70 µm, and the total thickness was 100 µm. Oxygen permeability was 25 cm^3^ m^−2^ 24 h^−1^ at a pressure of 0.1 MPa, relative humidity of 50 %, and a temperature of 25 °C. Water vapour permeability was 25 g m^−2^ 24 h^−1^ at a temperature of 38 °C, at the atmospheric pressure, and at a relative humidity of 90 %. Each packaging contained approx. 2 kg of crisps, therefore the number of replications for each version of crisps was *n* = 20. A packaging with the crisps was placed in the packing machine, and the pressure was reduced to the value of 8000 Pa (first step). Having reached this pressure, the pressure, inside the packing machine and the same in the bag with crisps, was recreated to the value of 90,000 Pa (second step) using the atmospheric air or argon supplied from a cylinder. Compressed argon with a purity of 99.9 was used. The packaging’s were heat-sealed and stored for 6 months under protection from light at room temperature (25 ± 2 °C) and a relative humidity of 45–60 %. The samples were stored in cupboards in the corridor of the laboratory. Sensory analysis and chemical analyses were performed immediately after the production of crisps, and after a 6-month period of storage. All types of corn crisps samples were stored with ten repetitions (*n* = 10).

### Sensory Analysis

The detailed sensory assessment of the samples of corn crisps was performed with implementation of the quantitative descriptive analysis (QDA) according to a procedure described by Stone and Sidel [25]. This way of sensory profiling was chosen as a method widely used for evaluation of different food products providing information about the whole product profile which can be easily analyzed as well as statistically and graphically presented [26–29]. The analytical procedure of sensory evaluation was conducted in accordance with ISO standard 1399 [30], in the Laboratory of Sensory Analysis of food4good Innovative Food Cluster.

### Sample Preparation and Presentation

The individual samples of each type of corn crisps (weighing around 15 g) were placed in transparent, odorless, plastic boxes (125 ml) covered with lids in order to contain the aroma uniformly in the sample’s headspace. The samples were evaluated at room temperature (22 °C). Unsweetened tea (60 °C) was used between samples as a neutralizer. The samples sets for each evaluator were coded individually with three-digit numbers to avoid the carry—over effect (the influence of the previous sample on the evaluation of the further one) the samples were presented randomly and the order of samples in the first session was different than in the second one.

### Assessors Selection and Training

The assessors engaged in sensory evaluation of corn crisps samples were chosen from sensory experts trained in accordance with ISO standard 8586-2 [31] and having broad experience in different food products. Firstly 15 evaluators participated in five preliminary sessions and performed according to ISO standards 1399 [30], during which they assessed corn crisps samples with and without addition of refined linseed oil. Within these meetings the list of 20 sensory descriptors together with detailed definitions was established using the consensus method []. During ten further training sessions sensory experts evaluated corn crisps samples prepared as it was described above^32^. Finally, ten assessors (five women and five men) at the age ranging from 39 to 51 were selected on the basis of their repeatability (*P* > 0.05) and discriminating capability (*P* < 0.30).

### List of Sensory Descriptors

During training sessions sensory experts figured out the lexicon of 20 sensory descriptors together with specific and univocal definitions. It included characteristics of odor (fatty, flaxen/grassy, rancid, burnt, pungent, fish, another), crispness, dryness, hardness, shape, yellow color, flavor (fatty, flaxen/grassy, burnt, bitter, pungent, fish, another) and overall sensory quality (overall quality—oq). Overall sensory quality in this case means harmony in odor, color, and flavor intensity without negative aftertastes such as bitter or flaxen/grassy. The overall sensory quality was measured at the end of individual sections realized by sensory expert but always with comparison to a control sample. This way we were able to get an answer which samples were similar or totally different from the control sample. A continuous non-structured 10-cm long scale with the left end corresponding to the lowest intensity (value 0) and the right end to the highest intensity (value 10) of the evaluated sensory attributes was used. Moreover overall sensory quality was assessed as not harmonized (value 0) and very harmonized (value 10).

### Testing Conditions

The evaluation was performed in a laboratory fulfilling the requirements of ISO standard 8589 [33] and equipped with separate booths illuminated with white light. ANALSENS NT version 4.0 (Caret Systemy Cyfrowe i Oprogramowanie Sp. z o. o., Gdańsk, Poland) for sessions planning, generation of random numbers for samples coding, collecting the individual results and the preliminary data processing was used. The evaluations were performed in the Laboratory of Sensory Analysis of food4good Innovative Food Cluster. Moreover the protocol of ethical and professional practices for the sensory analysis of food was used according to IFST Guidelines for Ethical and Professional Practices for the Sensory Analysis of Foods (http://www.ifst.org).

### Sessions in Sensory Evaluation

Two independent sessions separately for samples of corn crisps directly after production and after 6 months of storing these samples were performed. Each sample was assessed separately in two independent repetitions by an expert panel that consisted of ten members. The 20 individual results were taken into consideration for assessment of each trait.

### Determination of ALA in Corn Crisps

The content of ALA was determined in accordance with the procedure described in Szterk *et al*. [19].

A portion (50 ± 5 mg) of the extracted fat (following the previous extraction of fat from the corn crisps, employing the method of Folch, Lees and Sloane [34] was saponified with 0.5 N methanolic sodium hydroxide and methylated with 14 % BF_3_-methanol solution (boron trifluoride-methanol solution). The resulting methyl esters were extracted with heptane containing an internal standard (methyl undecanoate). Fatty acid methyl/ethyl esters were analyzed on the Trace GC Ultra gas chromatograph (Thermo Fisher Scientific) coupled with the ITQ Series GC-Ion Trap MS detector. Chromatographic separation was carried out on an RT2560 (RESTEK) capillary chromatography column. Temperature program: 2 min–80 °C isothermal, 1.5 °C min^−1^ to 230 °C temperature gradient, 15 min–230 °C isothermal, after the time returned to the beginning condition. Gas flow: linear velocity at 1 ml min^−1^, helium gas was used as the carrier gas, split ratio 1:100; injector temperature 240 °C. All types of corn crisps samples were analyzed in ten repetitions (*n* = 10).

### Determination of Peroxide Value

The peroxide value was determined following the previous extraction of fat from the corn crisps, employing the method Folch *et al*. [34]. The peroxide value was determined in accordance with AOAC Official Method 965.33 Peroxide Value of Oils and Fats [35], with the result being expressed in mg O_2_ kg^−1^ of corn crisps. All the types of corn crisps samples were analyzed in ten repetitions (*n* = 10).

### Statistical Analysis

The obtained results were analyzed statistically using Statistica 10 software (StatSoft Inc. 2012). Mean values and standard deviations (SD) were calculated using Excel software in MS Word Office. The following statistical methods were employed: hierarchical and non-hierarchical analysis, one way variance analysis (ANOVA) and principal component analysis (PCA) [36–39].

Hierarchical analysis involved use of the Ward agglomeration method, and the squared Euclidean distance was employed as the measure of distance. Criteria for the number of clusters were determined based on the course of the agglomeration process, and cluster significance was determined using one-way analysis of variance at *α* = 0.05 and the least significant difference test (LSD). Hierarchical analysis was the basis for non-hierarchical analysis using a certain number of significant clusters. PCA using Pearson correlation with application of the mean values of assessors. The matrix data set was composed of 17 rows related to corn crisps samples and 20 columns related to sensory traits.

## Results and Discussion

According to the employed method of profile assessment the following group of significant quality characteristics for corn crisps rich in α-linolenic acid was determined: o. fatty, o. flaxen/grassy, o. rancid, o. burnt, o. pungent, o. fish, o. another, crispness, dryness, hardness, shape, yellow color, f. fatty, f. flaxen/grassy, f. burnt, f. bitter, f. pungent, f. fish, f. another and overall quality.

**Fig. 1:**
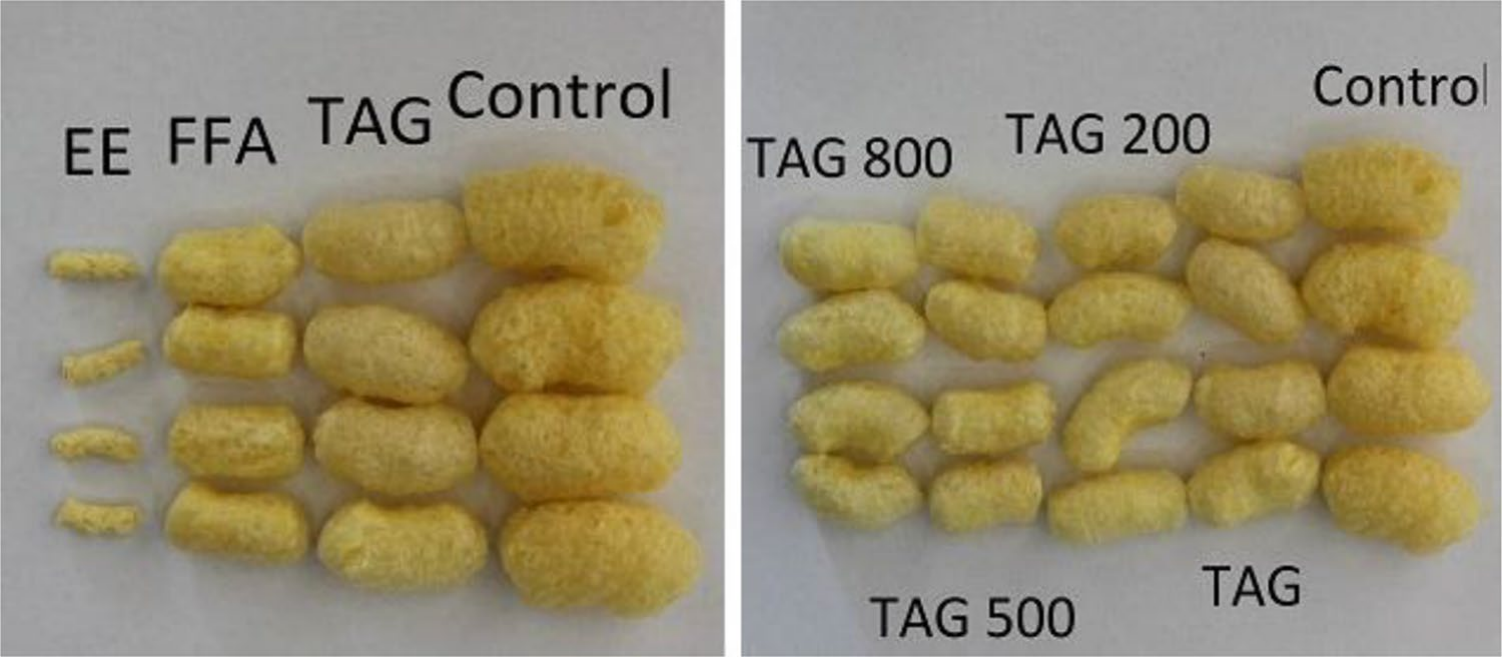
Corn crisps after 6 months of storage under atmospheric conditions obtained under the same parameters of the extrusion process from maize grit

Figure 1 presents the photographs of corn crisps after 6 months of storage in barrier packages (gas and sunlight blocking) filled with atmospheric air. It was observed that the crisps differ in size and color to a small extent. The biggest crisps do not contain the addition of refined linseed oil (control samples). The 5 % addition of linseed oil to the corn grit and 5 % of the same oil with δ-tocopherol in the amount of 200, 500 and 800 mg 100 g^−1^ to the corn grit resulted in a decrease in the size of the crisps. It has to be noted that δ-tocopherol did not affect the size of the product.

The use of linseed oil preparations in the form of free fatty acids and ethyl esters of these fatty acids significantly affected the size (level of expansion) of the corn crisps obtained.

**Fig. 2:**
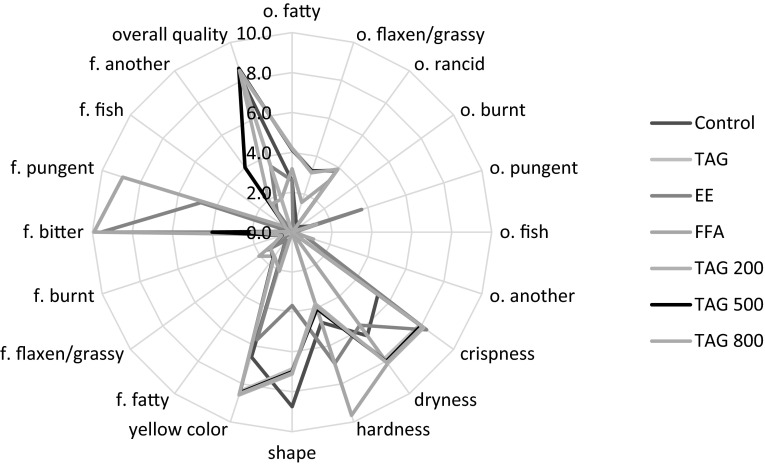
The qualitative sensory profile of various studied corn crisps, *o.* odor, *f.* flavor, *n* = 20

**Fig. 3:**
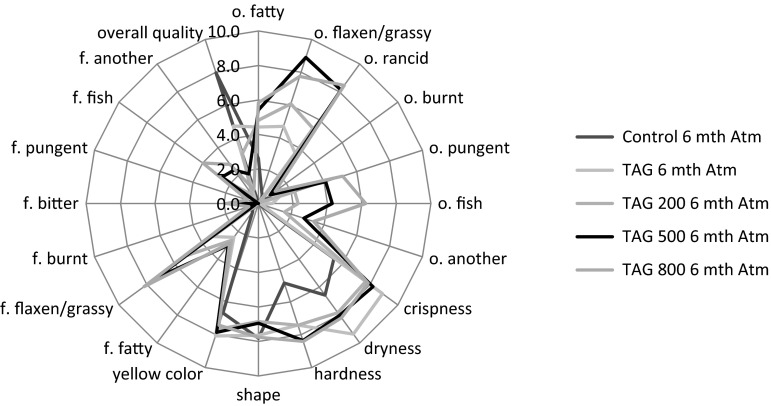
The qualitative sensory profile of various corn crisps after 6 month storage under atmospheric atmosphere, *o.* odor, *f.* flavor, *n* = 20

**Fig. 4:**
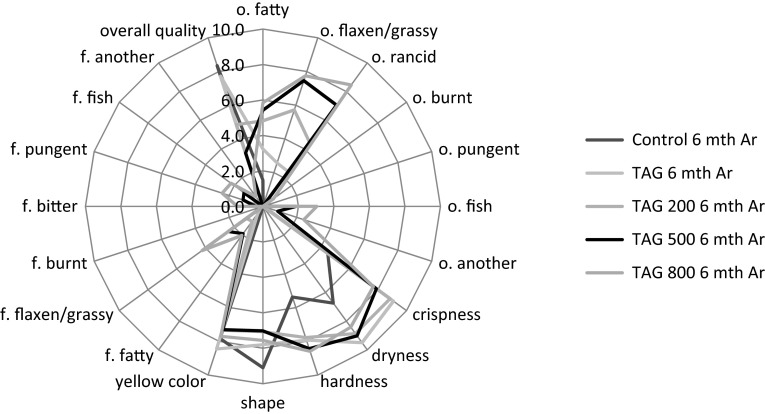
The qualitative sensory profile of various corn crisps after 6 month storage under 100 % argon atmosphere, *o.* odor, *f.* flavor, *n* = 20

Figures 2, 3 and 4 contain the quantitative and qualitative sensory profiles of the studied corn crisps. Based on the conducted research, it was concluded that in the sensory profile of the studied crisps the primary smell was the smell of grass/flax and a rancid smell. The second most important group of characteristics was related to physical properties such as crispness, dryness, hardness, shape, and yellow color. Among the significant properties were the grassy/flaxen flavor as well as the fish taste which were noticed after 6 months of storage. A similar phenomenon occurred in the case of burnt, pungent, fish and other smells. Other predictors constituted a small part of the qualitative and sensory profile. The intensity of all the qualitative characteristics differed significantly depending on the chemical composition of corn crisps and storage conditions.

**Fig. 5:**
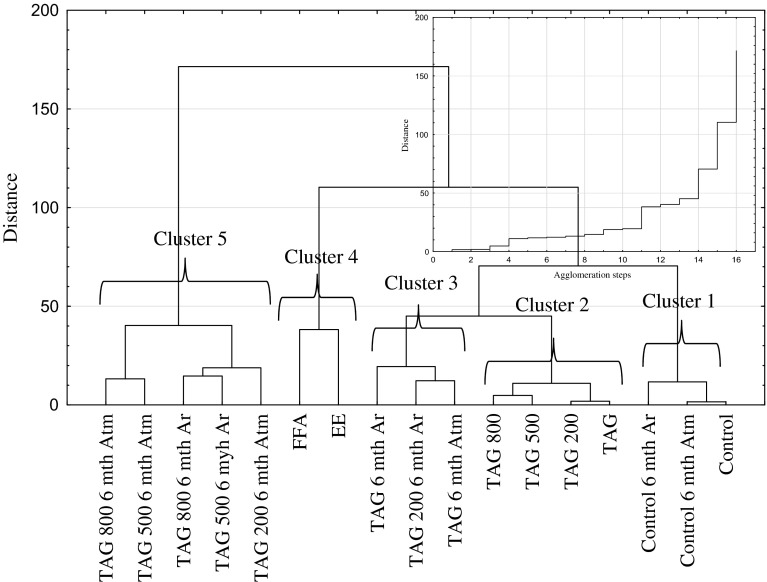
Hierarchical analysis of the sensory profile in different corn crisp samples. The actual *n* = 20 (the number of samples included in the chart) was reduced in order to obtain a an easily readable picture

Due to complex quantitative sensory profiles and a large number of examples, the data was subjected to hierarchical and non-hierarchical analysis which allowed to find samples similar to each other in terms of the sensory profile. Figure 5 presents the results of the analysis of these analyses using Ward’s method as well as the course of the agglomeration process. The hierarchical analysis revealed five clusters below the linkage distance of 40. The criteria for the number of clusters were defined based on the course of the agglomeration process which is also presented on Fig. 5. The critical value of linkage distance is the value at which the agglomeration progress starts to increase rapidly. It was assumed that the appropriate critical value was the linkage distance of 40. The cluster significance was established using single-factor variance analysis with *α* = 0.05 and applying the least significant difference test (LSD). It was concluded that four clusters were significantly different from each other (*P* < 0.05). There is no statistical difference between cluster 2 and cluster 3 (*P* > 0.05).

The first group of corn crisps similar in terms of their sensory profile were crisps manufactured using only corn grit without the addition of lipid fractions. These samples, which were stored for 6 months both in atmospheric air as well as in an atmosphere of argon, are similar to the freshly produced ones. The second group includes crisps initially containing a 5 % addition of refined linseed oil and 5 % refined linseed oil with various additions of δ-tocopherol (TAG, TAG 200–800). The third group contains corn crisps having a similar sensory profile to the ones present in the second group. In that case the corn crisps were enriched with 5 % refined linseed oil and were stored in atmospheric air and in an atmosphere of argon for 6 months (TAG 6 mth Ar and TAG 6 mth Atm). Moreover, this exact group also contained crisps having a 5 % addition of linseed oil with the addition of δ-tocopherol with an amount of 200 mg 100 g^−1^ which were stored for 6 months in an atmosphere of argon (TAG 200 6 mth Ar). Group 4 comprised corn crisps with a 5 % addition of ethyl esters of linseed oil fatty acids or 5 % free fatty acids obtained from the same oil. The last, fifth group consisted of corn crisps which were stored for 6 months both in atmospheric air and an atmosphere of argon and contained a 5 % addition of refined linseed oil with the addition of δ-tocopherol in various amounts.

**Fig. 6:**
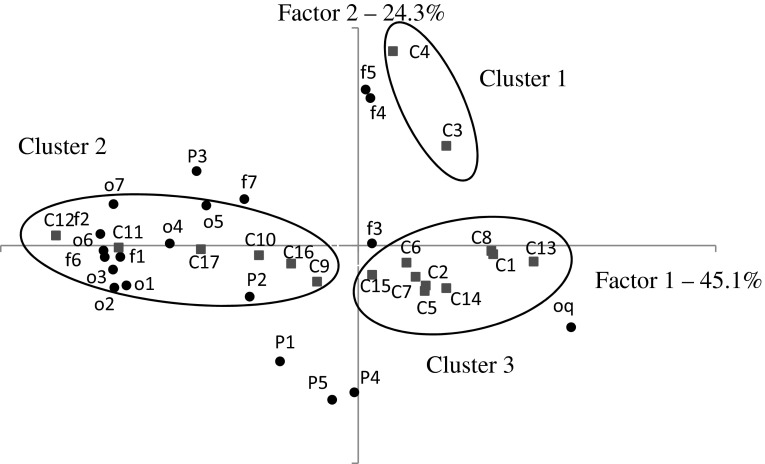
Principal component analysis plot of the similarities and differences in the sensory profiling characteristics of the corn crisps. *Black dots* represent sensory factors where: *o* odor, *P* some physical cases, *f* flavor, *o1* o. fatty; *o2* o. flaxen/grassy; *o3* o. rancid; *o4* o. burnt; *o5* o. pungent; *o6* o. fish; *o7* o. another; *P1* crispness; *P2* dryness; *P3* hardness; *P4* shape; *P5* yellow color; *f1* f. fatty; *f2* f. flaxen/grassy; *f3* f. burnt; *f4* f. bitter; *f5* f. pungent; *f6* f. fish; *f7* f. another; *oq* overall quality. *Red squares* represent studied cases: *C1* Control; *C2* TAG; *C3* EE; *C4* FFA; *C5* TAG 200; *C6* TAG 500; *C7* TAG 800; *C8* Control 6 mth Atm; *C9* TAG 6 mth Atm; *C10* TAG 200 6 mth Atm; *C11* TAG 500 6 mth Atm; *C12* TAG 800 6 mth Atm; *C13* Control 6 mth Ar; *C14* TAG 6 mth Ar; *C15* TAG 200 6 mth Ar; *C16* TAG 500 6 mth Ar; *C17* TAG 800 6 mth Ar (color figure online)

The data provided in Figs. 2, 3 and 4 were subjected to principal component analysis in order to find the most important qualitative characteristics that affect the sensory properties of functional corn crisps. Figure 6 presents the results of PCA, depicting the projection of cases and variables on the factor plane. The sensory quality of corn crisps enriched with linseed oil can be described using two groups of properties which include the majority of studied variables. The first group, which explains the variability of data set in over 45 %, includes the characteristics connected with the flavor and the smell of crisps. The second group of characteristics defining the quality of the studied crisps, explaining the data variability in over 24 %, is mostly connected with the physical properties (crispness, dryness, hardness, shape, yellow color). This group also includes bitter and pungent flavors. Figure 6 presents three clusters of cases. The first group of cases visible on the chart depicts corn crisps manufactured from corn grit with a 5 % addition of ethyl esters from linseed oil (C3) and 5 % of free fatty acids obtained from the same linseed oil (C4). The sensory profile of these corn crisps was dominated by bitter and pungent tastes which was not distinctive in other crisps. The second cluster of cases includes corn crisps which possessed a sensory profile characterized by a significant presence of such qualitative characteristics as smell: fatty, grassy/flaxen, rancid, pungent, fish-like, others (the majority of sensory panel members described it as spoiled) and then dryness, hardness and flavor: fatty, grassy/flaxen, fish-like other (the majority of sensory panel members described it as not fresh). The third cluster includes cases (samples) which possessed a sensory profile with a significantly lower share of the previously mentioned predictors and in addition were characterized by high values for yellow color (P5), crispness (P1), shape (P4), overall quality (oq) and low values for the burnt flavor (f3). The samples included in this cluster may be described as better than the others as their sensory profiles were similar to those of control samples as well as crisps containing 5 % of refined linseed oil.

**Fig. 7:**
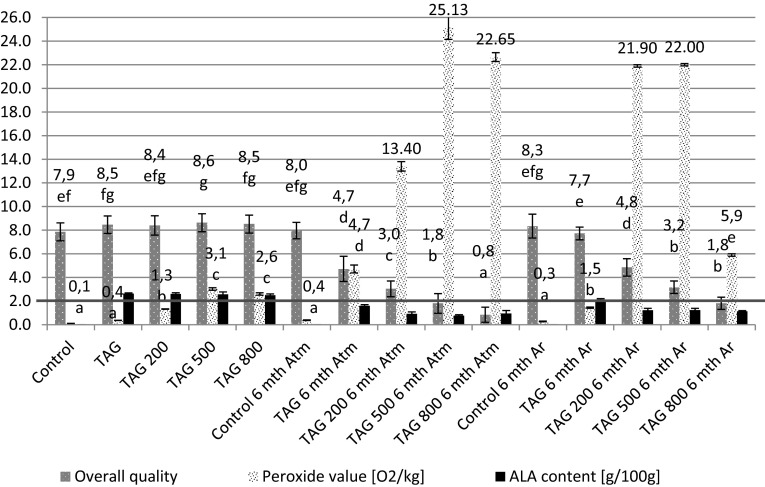
Overall quality (*n* = 20), concentration of α-linoleic acid—ALA (*n* = 10) and peroxide value (*n* = 10) in studied corn crisp samples after and before 6 month of storage during various conditions

An attempt was made to establish the correlation between the studied characteristics and the overall quality of the crisps, which might be a useful parameter allowing for quick and objective assessment of sensory similar to the control sample of corn crisps in relation to e.g. their chemical composition. As the research has demonstrated, the overall quality of corn crisps is a sum of selected sensory descriptors. It includes the flavor, smell and color. Figures 1–19 (supplementary materials) depict the influence of particular quality characteristics on the overall quality of the studied corn crisps. The majority of descriptors describing the flavor of crisps enriched with linseed oil which is a source of ALA is unfavorably correlated with the overall quality. Therefore, the lower numerical values are attributed to these sensory characteristics, the higher is the overall quality of the product. The exception to this tendency are variables such as crispness, shape, yellow color, and burnt flavor, for which high marks in the sensory panel are desired. It can be assumed that the overall quality of corn crisps is a good parameter differentiating the influence of various factors on the quality of corn crisps. It can be used for quick comparison of sensory properties of corn crisps and relate it to e.g. its chemical composition.

Figure 7 juxtaposes the overall quality of the studied corn crisps together with selected chemical parameters such as the amount of α-linolenic acid as well as the peroxide value. After the analysis of data presented in Fig. 7 it can be concluded that they overlap to a significant extent with the hierarchical and non-hierarchical analysis (Fig. 5) but they enable to establish which samples are the best sensory-wise. Owing to the above described procedure it is possible to relate the tastiness of corn crisps to their chemical composition which determines its classification as a functional food.

The conducted experiments demonstrate that crisps manufactured from corn grit without the addition of lipid fraction are characterized by a similar overall quality (harmony in odor, color and flavor intensity without negative aftertastes such as bitter or flaxen/grassy odor and flavor and so on) to freshly produced crisps with a 5 % addition of refined linseed oil and 5 % of the same oil with various additions of δ-tocopherol (200–800 mg 100 g^−1^). Storing corn crisps for 6 months in packages filled with argon or atmospheric air in most cases leads to deterioration of their overall quality. Only control samples (without the addition of lipid fraction) stored both in argon and atmospheric air as well as TAG 6 mth Ar sample do not differ from the freshly produced samples—they belong to one homogenous group. Other samples are characterized by significantly lower marks which causes them to be deemed as less tasty or not tasty since their characteristic sensory profile resulted in their low overall quality score. By relating the overall quality of corn crisps to the peroxide value it can be concluded that the higher it is, the more unfavorable was the assessment of the crisps by the sensory panel experts. A high peroxide value not only leads to low overall quality, but it also causes a decrease of the amount of ALA, which is presented on Fig. 7. The degradation of ALA acid leads to a decrease of its concentration in the final product. A decrement of ALA below 2 g 100 g^−1^ (a daily portion) prevents a product from being classified as functional. The conducted study showed that corn crisps which were enriched in 5 % with refined linseed oil and stored for 6 months in barrier packages (gas and sunlight blocking) filled with argon may receive high score in terms of overall quality (similar to the input value) and sensory profile, which is similar to that of a control sample as well as the 6-month-old crisps (Figs. 5, 6). Moreover, these crisps contain over 2 g of ALA per 100 g in the final product which allows them to be classified as functional food (Fig. 7).

According to the regulation No. 432/2012 of the European Commission of 16 May 2012 [20], the following health claims can be placed on a food product: “α-Linolenic acid contributes to the maintenance of normal blood cholesterol levels” and “this product is a source of omega-3 fatty acids” only if that product contains 2 g of ALA per portion so that it is possible to provide 2 g of α-linolenic acid to the body through consumption of the product. In order to comply with the regulation of European Commission No. 432/2012 [20] it was decided to make a 5 % addition of linseed oil, which is a very good and valuable source of ALA, to the production formula of corn crisps. The used linseed oil contains on average 57–58 % of ALA, meaning that its addition in the amount of 5 % enables to obtain at least 2.85 g of ALA in a 100 g portion [19, 21]. Apart from the chemical composition, a very important aspect of a functional product is its taste/flavor. In order to successfully introduce a product into the market and to ensure that it serves its biological functions it has to be asserted that the product tastes similar to control sample. The flavor of a product has to remain unchanged not only just after its manufacturing but also after its storage in warehouses or store shelves. The majority of studies relating to the content of various chemical substances (including biological substances) in extruded products and their influence on sensory properties is almost exclusively restricted to studying these types of products right after their manufacturing. In many cases the studies are only concerned with the influence of extrusion parameters on a product’s sensory properties [40–45]. Other works focus on the influence of various recipe additions on sensory properties as well as the health-beneficial qualities of extruded products, e.g. the influence of buckwheat [46]; oats, which are a valuable source of β-glucans [47], fibre from apple pomace [48], wheat bran [49], whey or soy proteins [50–53], fish protein [54], dried shrimps [55], fish oil [56], various dried vegetables [57–59], seaweed [60], mineral ingredients [53], hemp seeds [61] or flax seed [62], however without conducting sensory research during the storage of an extruded product. It is known that during the storage of extruded products characterized by low water activity, a developed interphase contact surface and containing polyunsaturated fatty acids as well as other active biological substances, various unfavorable oxidation processes will occur, leading to a changes in taste of a food product [22].

Our previous works [19, 21] described the chemical and textural properties of corn crisps, which could be classified as functional food products containing omega-3 fatty acids. However, the optimum chemical composition is not a guarantee of success. Another very important aspect is sensory research which would confirm whether the designed functional product can be a source of health-beneficial substances under real conditions. As the conducted experiments have demonstrated (Figs. 2, 6), corn crisps with the addition of two fatty preparations: ethyl esters and free fatty acids obtained from refined linseed oil are not suitable for commercial production since they are bitter and pungent in taste. Therefore, their overall sensory quality is very low in relation to the control sample, which serves as a benchmark in this regard—all the manufactured crisps should be on a comparable level or surpass it in terms of overall sensory quality (Fig. 7). Moreover, these crisps are characterized by a low level of expansion, causing them to be smaller and harder, which is not a favorable characteristic of their quality (Fig. 1). The usage of these linseed oil preparations had a purpose of increasing the stability of ALA during storage. The studies led by Morrison *et al*. [63] and Nuessli *et al*. [64] indicate the fact that ethyl esters of fatty acids as well as free fatty acids in extruded starch products may display a larger oxidation stability since they react with amylose chains surrounding it, which in this way protect it against radicals or reactive oxygen species. Unfortunately, this form of introducing a lipid fraction into extruded crisps makes it impossible to produce them using the most widespread technology—corn grit extrusion using a single screw extruder. The use of refined linseed oil for functional crisps production allowed us to obtain a sensory profile and overall quality very similar to those of control samples (crisps without the addition of lipid fraction), which in this case serve as a benchmark—all the manufactured crisps should be on a comparable level or surpass them in terms of quality (Figs. 5, 7). The use of refined linseed oil had the purpose of removing the characteristic “grassy” flavor of the linseed oil itself as well as its bitterness, which results from the presence of cyclolinopeptides (Aladedunye *et al*. [65]). After the analysis of data shown in Figs. 2, 3 and 4 it can be concluded that this goal has been achieved. The obtained corn crisps did not have a bitter flavor except for the EE and FFA samples, the bitter flavor of which was most probably not a result of the presence of cyclolinopeptides but the reaction of fatty preparations with starch and other components of corn grit, which led to a change of flavor.

In order to increase the oxidation stability of ALA as well as the whole lipid fraction, one of the strongest natural-based antioxidants is δ-tocopherol, which is a more active antioxidant than α-tocopherol, and this was used [66, 67]. The manufactured crisps were additionally packed in barrier packaging (gas and sunlight blocking) filled with either 100 % argon or atmospheric air. The conducted research showed that the sensory properties of crisps change significantly during their storage. As a result of ongoing oxidation processes, their flavor deteriorates over time [20]. The addition of δ-tocopherol did not bring the expected result. The deterioration of flavor in crisps with the addition of δ-tocopherol was caused by the pro-oxidative effect of this compound. The pro-oxidative effect of tocopherols was studied and explained by Rogalski and Szterk [21]. The unfavorable chemical reactions occurring in corn crisps’ lipid fraction led to oxidation of polyunsaturated and monounsaturated fatty acids. The formed aldehydes, short-chain fatty acids, alcohols, ketones etc. were responsible for the intensification of fatty, grassy, rancid smell and flavor as well as the deterioration of physical properties, including the color (Figs. 2, 3, 4). Moreover, the oxidation processes resulted in a taste and smell classified as “other”, which were respectively described as “not fresh” and spoilt. In general, the overall quality of the product has deteriorated. Unfortunately, in some food products, especially those rich in ions of transition metals and those characterized by low water activity, there is no possibility of using antioxidants since they may have a pro-oxidative effect [21, 22]. In this case, the increase in chemical stability can be obtained by using physical procedures, e.g. using a modified atmosphere or modifying the technological process. Modification of the technological process is not always possible, especially in the case when the extrusion is done using single screw extruders. The conducted studies demonstrated that increasing the chemical and sensory stability of corn crisps enriched with n-3 fatty acids through using various linseed oil preparations and adding tocopherols as antioxidants did not bring satisfactory results. However, it is possible to manufacture a product which would be stable for 6 months of storage in room temperature if the corn crisps containing a 5 % addition of linseed oil were stored in barrier packaging (sunlight and gas blocking) filled with 100 % argon. Similar results were obtained in the research by Shaviklo *et al*. [54], who studied oxidation changes as well as the sensory properties of corn crisps enriched with various fish proteins. Their study did not demonstrate any changes in the qualitative and quantitative sensory profiles of corn crisps after 6 months of their storage in oriented polypropylene (OPP) packages, even with exposure to sunlight and atmospheric air. They concluded that there was no need of using antioxidants in order to obtain a high sensory quality of crisps enriched with fish protein. Similar results were also obtained in a study concerning the chemical and sensory stability of corn crisps with the addition of shrimps (which are also a source of n-3 acids) conducted by Shaviklo *et al*. [55]. They also observed a high oxidation and sensory stability in corn crisps containing as much as 28 % of fatty fraction without the addition of antioxidants, which were stored for 90 days in OPP packages filled with atmospheric air. Rababah *et al*. [68] studied the oxidation stability as well as quantitative and qualitative changes in the sensory profile of corn crisps enriched with grape seed extract. Their research showed that the addition of synthetic antioxidants (BHT 200 ppm) as well as the addition of grape seed extract in the amount of 400 and 800 ppm contribute to the significant increase of chemical quality and preservation of the original qualitative and quantitative sensory profile of corn crisps containing approximately 5 % of fatty fraction. The storage of corn crisps without the addition of antioxidants in packages filled with atmospheric air contributed to the oxidation of lipid fraction, leading to the deterioration of the sensory profile. Similarly to our research, they also observed the increase in the percentage share of rancid taste and smell as well as the deterioration in the overall quality. Our research demonstrated that corn crisps enriched in 5 % with linseed oil are stable for 6 months in barrier packaging filled with argon. Their performance in packages filled with atmospheric air is significantly worse and in this regard the results of our studies differ from those of Shaviklo *et al*. [54, 55], but at the same time, they are concordant with the research by Rababah *et al*. [68]. The usage of argon as the atmosphere modifier in the packaging is caused by the fact that it is almost completely chemically passive, creating only unstable hydrates ${\text{Ar}} \cdot 6 {\text{H}}_{ 2} {\text{O}}$. By removing the oxygen from the package, all the oxidation processes are effectively reduced. Moreover, due to its large molecular mass it is significantly easier to retain it within the package. There is no defined dosage of argon which is allowed for usage in the food industry. In addition, it is the cheapest noble gas of all. Nitrogen or a mix of nitrogen with e.g. argon is typically used for packaging. However, nitrogen is significantly more chemically reactive in comparison to argon and may, for instance, be subject to free-radical reactions occurring in the oxidized lipid fraction, especially in dried product such as corn crisps, where a loose contact of nitrogen molecules is allowed since there is no water which would restrict this contact and reduce the kinetics of auto-oxidation [18, 22, 69].

## Conclusion

It is possible to manufacture functional corn crisps rich in n-3 fatty acids of plant origin. Using refined linseed oil for manufacturing corn crisps while applying the traditional technology of their production and packing them in barrier packages filled with argon enables one to obtain a product which is a source of ALA even after 6 months of storage and maintains a similar—the quantitative sensory profile is relatively unchanged in comparison to the freshly manufactured product as well as the crisps without the addition of linseed oil. A daily calorie requirement for women aged 18–54 is approximately 2200 kcal (non-strenuous work) and for men in similar age it is approximately 2200 kcal (non-strenuous work). An addition of 100 g of corn crisps containing approx. 360 kcal, which are also a source of at least 2 g of ALA would constitute respectively 16 and 14 % of the daily calorie requirement. By including functional corn crisps in a balanced daily diet it is possible to effectively supply omega-3 fatty acids through the consumed food. In addition, elderly people and children often consume extruded corn crisps as a snack. Replacing them with very similar crisps rich in ALA will bring a positive result of supplying this valuable fatty acid known for its numerous health-beneficial effects to the body. The increase in consumption of deficient n-3 fatty acids will help to reduce the occurrence of diseases of civilization such as cardiovascular diseases which are a result of excessive concentration of cholesterol in the blood. Functional corn crisps may be a very good alternative for people who already consume extruded snacks as they increase the nutritional value of a daily food ration.

## Electronic supplementary material

Below is the link to the electronic supplementary material.

Supplementary material 1 (DOCX 490 kb)

## Abbreviations

ALA: α-Linolenic acid
BHT: Butylhydroxytoluene
DHA: Docosahexaenoic acid
DPA: Docosapentaenoic acid
EPA: Eicosapentaenoic acid
OPP: Oriented polypropylene
PA/PE: Polyamide/polyethylene
QDA: Quantitative descriptive analysis
TAG: Triacylglycerol
RLO: Refined linseed oil
