# Supplementary material for: Corn Crisps Enriched in Omega-3 Fatty Acids Sensory Characteristic and its Changes During Storage
Source: J Am Oil Chem Soc. 2016 Jul 23;93(9):1275–87. doi: 10.1007/s11746-016-2873-y (PMC5010839; doi:10.1007/s11746-016-2873-y)
Supplement: Supplementary file 1 — Supplementary material 1 (DOCX 490 kb) [file 11746_2016_2873_MOESM1_ESM.docx]

Fig. A.1 Odour of fatty vs overall quality

Fig. A.2 Odour of flaxen/grassy vs overall quality

Fig. A.3 Odour of rancid vs overall quality

Fig. A.4 Odour burnt vs overall quality

Fig. A.5 Odour of pungent vs overall quality

fig. A.6 Odour of fish vs overall quality

Fig. A.7 Odour of another vs overall quality

Fig. A.8 Crispness vs overall quality

Fig. A.9 Dryness vs overall quality

Fig. A.10 Hardness vs overall quality

Fig. A.11 Shape vs overall quality

Fig. A.12 Yellow colour vs overall quality

Fig. A.13 fatty flavor vs overall quality

Fig. A.14 Flaxen/grassy flavor vs overall quality

Fig. A.15 Burnt flavor vs overall quality

Fig. A.16 Bitter flavor vs overall quality

Fig. A.17 Pungent flavor vs overall quality

fig. A.18 Fish flavor vs overall quality

Fig. A.19 Another flavor vs overall quality
